# Deletion of ACLY Disrupts Histone Acetylation and IL-10 Secretion in Trophoblasts, Which Inhibits M2 Polarization of Macrophages: A Possible Role in Recurrent Spontaneous Abortion

**DOI:** 10.1155/2022/5216786

**Published:** 2022-05-11

**Authors:** Xin Chen, Qian Lin Song, Ze Hong Li, Rui Ji, Jia Yu Wang, Chang Ge, Zhuo Ni Xiao, Duan Ying Guo, Jing Yang

**Affiliations:** ^1^Reproductive Medical Center, Renmin Hospital of Wuhan University and Hubei Clinic Research Center for Assisted Reproductive Technology and Embryonic Development, Wuhan, Hubei, China; ^2^Department of Urology, Renmin Hospital of Wuhan University, Wuhan, Hubei, China; ^3^Longgang District People's Hospital of Shenzhen, Shenzhen, China

## Abstract

Changes to macrophage polarization affect the local microenvironment of the placenta, resulting in pathological pregnancy diseases such as recurrent spontaneous abortion (RSA). Macrophages are in close contact with trophoblasts during placental development, and trophoblast-derived cytokines are important regulators of macrophage polarization and function. Histone acetylation can affect the expression and secretion of cytokines, and ATP citrate lyase (ACLY) is an important factor that regulates histone acetylation. The aim of this study was to investigate the effect of ACLY expression differences in trophoblast on macrophage polarization and its mechanism. Our data demonstrate that ACLY level in placental villi of patients with RSA is decreased, which may lead to the inhibition of histone acetylation in trophoblasts, thereby reducing the secretion of IL-10. Reduced IL-10 secretion activates endoplasmic reticulum stress in macrophages, thus inhibiting their M2 polarization.

## 1. Introduction

Throughout pregnancy, the polarity and function of macrophages play an important role in intrauterine immune homeostasis and can also affect the process of pregnancy by promoting spiral artery remodeling and regulating trophoblast invasion and apoptosis [1–3]. Therefore, the polarity and function of macrophages are very important for the maintenance of a normal pregnancy.

According to the different inducing factors, phenotypes, and functions, M *φ* macrophages can be divided into M1 and M2 macrophages, similar to Th1 and Th2 T helper cells. M1 cells usually have proinflammatory activity, while M2 cells have an anti-inflammatory role [4–6]. In the placenta of an early pregnancy, macrophages are mainly the M2 type, which is very important for normal pregnancy [7]. A decrease in the proportion of M2 macrophages or an increase in the proportion of M1 macrophages can lead to recurrent abortion [8]. Trophoblast-derived cytokines are very important to induce the balance and immune tolerance of local M1 and M2 cells in the uterus, thus maintaining a normal pregnancy [9]. The functional diversity of macrophages reflects the high plasticity of the microenvironment at the maternal-fetal interface [10]. Appropriate and timely regulation of M1 to M2 polarization is considered the key factor to establish and maintain pregnancy at different stages. However, an imbalance of this polarization will lead to disruption of the microenvironment at the maternal-fetal interface, leading to pathological pregnancy and diseases, such as recurrent spontaneous abortion (RSA) [11].

ATP citrate lyase (ACLY) is one of the main enzymes that catalyze the formation of cytoplasmic acetyl CoA. In addition to providing the classical function of acetyl CoA for new fat production, ACLY also participates in epigenetic regulation through histone acetylation [12]. There is evidence that epigenetic modifications are involved in early embryogenesis, and defects in epigenetic patterns contribute to the occurrence and development of RSA [13, 14]. Histone modification is one of the main epigenetic mechanisms [15]. In recent years, studies have found that histone acetylation plays an important role in the expression of cytokines [16]. For example, the application of histone deacetylase (HDAC) inhibitors, such as trichostatin A (TSA), leads to extensive acetylation of histones, thus reducing the expression of genes encoding inflammatory cytokines, such as interleukin- (IL-) 2, IL-6, and interferon- (IFN-) *γ*, as well as reducing the symptoms of lupus prone mice [17]. However, despite being a powerful epigenetic regulator, it is still unknown whether ACLY plays an important role in the maintenance of the maternal fetal-interface immune system, and whether ACLY participates in macrophage polarization and their functional regulation, thus affecting the pathogenesis of recurrent abortion.

In this study, we aimed to evaluate the localization and expression of ACLY in placental tissues of patients with a normal pregnancy and patients with RSA and to determine the role, if any, of ACLY in RSA. Through *in vitro* cell experiments, it was confirmed that knockdown of *ACLY* in trophoblasts led to the inhibition of histone acetylation, which reduced the secretion of IL-10 from trophoblasts. This reduction of IL-10 activated endoplasmic reticulum stress in macrophages, thus inhibiting their M2 polarization. Therefore, the results of the present study provide new insights and ideas for the pathogenesis of RSA.

## 2. Materials and Methods

### 2.1. Patient and Tissue Samples

A total of 25 patients with induced abortion due to accidental pregnancy (the healthy control, HC group) and 25 patients with RSA were included in this study. Patients with the following characteristics were excluded: (1) pelvic examination and ultrasound showing genital malformations, (2) chromosomal abnormality of the parent or embryo, (3) infectious diseases, and (4) symptoms of endocrine or metabolic diseases. Samples were collected immediately after curettage and washed with PBS. One part of the collected placental tissue was fixed in 4% paraformaldehyde and embedded in paraffin. The other part of the tissue was frozen and stored in liquid nitrogen. All samples were collected with the informed consent of the patients, and all relevant procedures were approved by the internal review and ethics committee of the Renmin Hospital of Wuhan University. The baseline characteristics of the patients are summarized in Table 1.

### 2.2. Immunofluorescence

Tissue sections were dewaxed, washed, subjected to antigen repair, and blocked. Primary antibodies recognizing cytokeratin 7 (CK7) (66483-1-Ig, 1 : 1,000 dilution, Proteintech, Wuhan, China), human leukocyte antigen-G (HLA-G) (66447-1-Ig, 1 : 200 dilution, Proteintech), and ACLY (15421-1-ap, 1 : 100 dilution, Proteintech) were added and incubated overnight at 4°C. After washing with PBST, horseradish peroxidase- (HRP-) labeled Goat anti rabbit (5220-0336, 1 : 1,000 dilution, SeraCare, Millford, MA, USA) or HRP-labeled Goat anti mouse (5220-0341, 1 : 1,000 dilution, SeraCare) secondary antibodies were added and incubated in the dark at room temperature for 50 min. Tyramine salt-CY3 or Tyramine salt-488 was added and incubated in the dark at room temperature for 20 min. The nuclei were stained using 4′,6-diamidino-2-phenylindole (DAPI) and then observed using an orthographic microscope system (Olympus BX51, Tokyo, Japan).

### 2.3. Immunohistochemistry

The tissue sections were dewaxed, washed, subjected to antigen repair, and blocked. Primary antibodies recognizing cytokeratin 7 (CK7) (66483-1-Ig, 1 : 2,000 dilution, Proteintech), human leukocyte antigen-G (HLA-G) (66447-1-Ig, 1 : 400 dilution, Proteintech), ACLY (15421-1-ap, 1 : 100 dilution, Proteintech), acetyl-Histone H3 (af4365, 1 : 100 dilution, Affinity Biosciences, Jiangsu, China), activating transcription factor 6 (ATF6) (24169-1-ap, 1 : 200 dilution, Proteintech), glucose-regulated protein, 78 kDa (GRP78) (11587-1-ap, 1 : 400 dilution, Proteintech), phosphorylated- (p-) PRKR-like endoplasmic reticulum kinase (PERK) (df7576, 1 : 200 dilution, Affinity Biosciences), p-inositol-requiring enzyme 1 (IRE1) (af7150, 1 : 200 dilution, Affinity Biosciences), and XBP1s (24868-1-AP, 1 : 400 dilution, Proteintech) were added and incubated overnight at 4°C. The sections were washed with PBST, and HRP-labeled Goat anti-rabbit (5220-0336, 1 : 1,000 dilution, SeraCare) or HRP-labeled Goat anti-mouse (5220-0341, 1 : 1,000 dilution, SeraCare) secondary antibodies were added to cover the tissue, followed by incubation at room temperature in the dark for 50 min. Diaminobenzidine (DAB) chromogenic solution was added, and the sections were observed under an upright microscope system (Olympus BX51).

### 2.4. Cell Culture

HTR-8 and JEG-3 cells were cultured in Dulbecco's modified Eagle's medium (DMEM)/F12 medium, JAR cells were cultured in DMEM medium, BeWo cells were cultured in minimal essential (MEM) medium, and THP-1 cells were cultured in Roswell Park Memorial Institute- (RPMI-) 1640 medium. All cells were cultured in a cell incubator containing 5% CO_2_ and sufficient humidity at 37°C.

### 2.5. Cell Transfection

Lentiviruses expressing a short hairpin RNA (shRNA) targeting *ACLY* or a control shRNA were synthesized by Shanghai GeneChem (Shanghai, China). The RNA interference (RNAi) target sequence was as follows: ACLY-RNAi-1 (CTAAGTACTCTTGCCAGTT), ACLY-RNAi-2 (TGAGAGCAATTCGAGATTA), and ACLY-RNAi-3 (AGGACTTGTACTTCACCTA). According to the manufacturer's instructions, the ACLY shRNA and control shRNA lentiviruses were transfected into cells, and the stably expressing cell lines were screened using puromycin.

### 2.6. THP-1 Differentiation and Coculture System

For macrophage polarization, THP-1 cells were incubated in 100 ng/ml phorbol 12 myristate 13 acetate (PMA, Sigma, St. Louis, MO, USA) for 24 hours to differentiate into M0 macrophages. To establish a coculture system, we cultured the M0 macrophages with culture supernatants of stably transfected and control HTR-8, JEG-3, and JAR cells as conditioned medium. After 72 hours of culture, the macrophages were collected for analysis.

### 2.7. RNA Isolation and Quantitative Real-Time Reverse Transcription PCR (qRT-PCR)

Total RNA was extracted using the TRIzol reagent (Invitrogen, Waltham, MA, USA) according to the manufacturer's instructions. After the RNA concentration and purity were detected, the PrimesScript ™ RT Master Mix (perfect real-time) (Takara, Shiga, Japan) was used to reverse transcribe the total RNA into cDNA. The cDNA was used as a template in a TB green ® Premix Ex Taq™ reaction to quantify the mRNA expression. *GAPDH* (encoding glyceraldehyde-3-phosphate dehydrogenase) was used as the internal reference. The primer sequences were as follows: *ACLY*, forward: 5′-GCTCTGCCTATGACAGCACCAT-3′, reverse: 5′-GTCCGATGATGGTCACTCCCTT-3′; *CD206*, forward: 5′-AGCCAACACCAGCTCCTCAAGA-3′, reverse: 5′-CAAAACGCTCGCGCATTGTCCA-3′; *CD163*, forward: 5′-CCAGAAGGAACTTGTAGCCACAG-3′, reverse: 5′-CAGGCACCAAGCGTTTTGAGCT-3′; *CCL18* (encoding C-C motif chemokine ligand 18), forward: 5′-GTTGACTATTCTGAAACCAGCCC-3′, reverse: 5′-GTCGCTGATGTATTTCTGGACCC-3′; CXCL16 (encoding C-X-C motif chemokine ligand 16), forward: 5′-CCTATGTGCTGTGCAAGAGGAG-3′, reverse: 5′-CTGGGCAACATAGAGTCCGTCT-3′; *IL10*, forward: 5′-TCTCCGAGATGCCTTCAGCAGA-3′, reverse: 5′-TCAGACAAGGCTTGGCAACCCA-3′; *IL6*, forward: 5′-AGACAGCCACTCACCTCTTCAG-3′, reverse: 5′-TTCTGCCAGTGCCTCTTTGCTG-3′; *IL-34*, forward: 5′-CCAAGGTGGAATCCGTGTTGTC-3′, reverse: 5′-CACCTCACAGTCCTGCCAGTTT-3′; *MCSF* (encoding macrophage colony stimulating factor), forward: 5′-TGAGACACCTCTCCAGTTGCTG-3′, reverse: 5′-GCAATCAGGCTTGGTCACCACA-3′; *GAPDH*, forward: 5′-GTCTCCTCTGACTTCAACAGCG-3′, reverse: 5′-ACCACCCTGTTGCTGTAGCCAA-3′.

### 2.8. Flow Cytometry

After sample treatment, a single cell suspension was collected and incubated with Human TruStain FcX™ (BioLegend, San Diego, CA, USA) at room temperature for 10 minutes; then, a phycoerythrin- (PE-) coupled anti-CD206 antibody (BioLegend) was added, incubated at 4°C for 20 minutes, and washed twice with cell staining buffer (BioLegend). 7-Aminoactinomycin D (7-AAD) Viability Staining Solution (BioLegend) was added and incubated in the dark for 5 minutes. The cells were immediately analyzed using flow cytometry (BD FASC Calibur, USA).

### 2.9. Enzyme-Linked Immunosorbent Assay (ELISA)

After sample collection, the level of IL-10 was determined using a human IL-10 ELISA kit (Cusabio, Wuhan, China) according to the manufacturer's instructions.

### 2.10. Western Blotting

Radio Immunoprecipitation Assay Lysis Buffer (P0013B, Beyotime, Shanghai, China) or EpiQuik Total Histone Extraction Kit (OP-0006-100, Amyjet Scientific, Wuhan, China) was used to extract total protein or total histone, respectively. The protein sampled was subjected to protein gel electrophoresis and then transferred onto a PVDF membrane. The membrane was then incubated with primary antibodies recognizing Histone-H3 (17168-1-AP, 1 : 5,000 dilution, Proteintech), acetyl-Histone H3 (af4365, 1 : 1,000 dilution, Affinity Biosciences, Jiangsu, China), ACLY (15421-1-ap, 1 : 2,000 dilution, Proteintech), GAPDH (60004-1-Ig, 1 : 50,000 dilution, Proteintech), activating transcription factor 6 (ATF6) (24169-1-ap, 1 : 1,000 dilution, Proteintech), glucose-regulated protein, 78 kDa (GRP78) (11587-1-ap, 1 : 2,000 dilution, Proteintech), phosphorylated- (p-) PRKR-like endoplasmic reticulum kinase (PERK) (df7576, 1 : 2,000 dilution, Affinity Biosciences), p-inositol-requiring enzyme 1 (IRE1) (af7150, 1 : 1,000 dilution, Affinity Biosciences), and XBP1s (24868-1-AP, 1 : 1,000 dilution, Proteintech). After washing with Tris-buffered saline-Tween 20 (TBST), the membrane was incubated in anti-rabbit secondary antibody (5151, 1 : 30,000 dilution, CST) or anti-mouse secondary antibody (5257, 1 : 15,000 dilution, CST) at room temperature for 1 hour and then washed with TBST. The immunoreactive proteins on the membrane were visualized using the Odyssey infrared imaging system (LI-COR, Lincoln, NE, USA), and then, the gray value of the protein bands was analyzed using the ImageJ software (NIH, Manassas, MD, USA).

### 2.11. Statistical Analysis

Data from at least three independent experiments were analyzed, and all data are expressed as the mean ± standard error (SD) of mean. All statistical analyses were performed using GraphPad Prism 6.01 (GraphPad Inc., La Jolla, CA, USA). Student's *t*-test was used to evaluate difference between two groups, and one-way ANOVA was used to compare difference between multiple groups. *p* < 0.05 was considered statistically significant.

## 3. Results

### 3.1. Localization of ACLY in Human Placental Tissue

**Figure 1:**
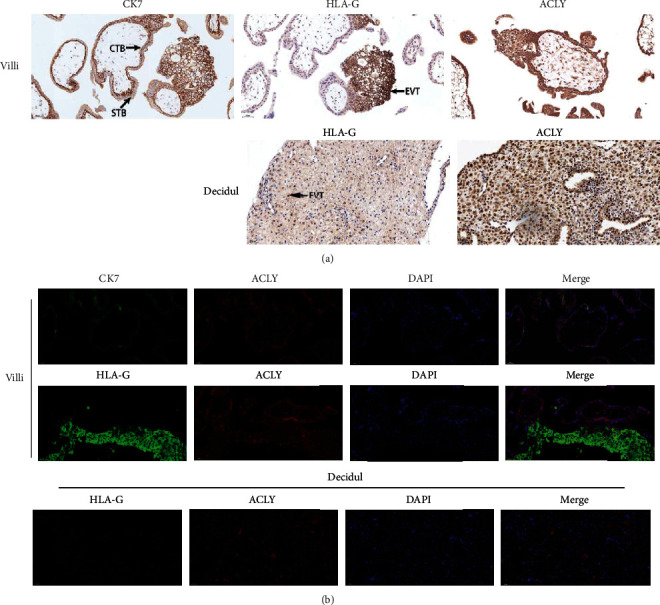
Location of ACLY in human placental tissue during early pregnancy. ACLY expression in cytotrophoblasts (CTBs), syncytiotrophoblasts (STBs), and extravillous trophoblasts (EVTs), as detected using immunohistochemistry (a) and immunofluorescence (b). Cytokeratin 7 (CK7) and human leukocyte antigen-G (HLA-G) were used as markers of CTBs and EVTs, respectively. Specific fluorescence staining of ACLY is stained red, CK7 or HLA-G is stained green, and the nucleus is stained blue (DAPI staining). All images are of a 200x visual field under the microscope. Scale bar = 50 *μ*m.

We first detected ACLY location in human placental tissue during early pregnancy using immunohistochemistry and immunofluorescence techniques, and all images were of a 200x visual field under the microscope (Figure 1). The results showed that ACLY was expressed in cytotrophoblasts (CTBs), syncytiotrophoblasts (STBs), and extravillous trophoblasts (EVTs) (Figures 1(a) and 1(b)).

### 3.2. ACLY Expression Is Decreased in Chorionic Tissue of Patients with RSA

**Figure 2:**
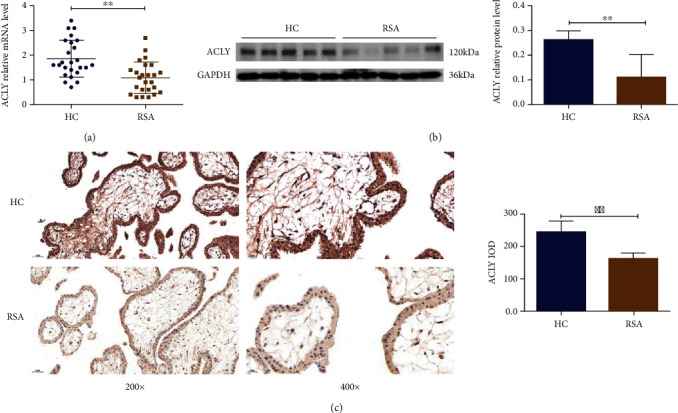
Decreased ACLY expression in chorionic tissue of patients with recurrent spontaneous abortion (RSA). (a) *ACLY* mRNA expression levels in the villi of patients with RSA and the healthy control (HC) group, as determined using qRT-PCR (*n* = 25). (b) The protein levels of ACLY in the villi of patients with RSA and the HC group, as analyzed using Western blot (*n* = 5). (c) The protein levels of ACLY in villi of patients with RSA and the HC group, as measured using immunohistochemistry (*n* = 5). ^∗∗^*p* < 0.01, compared with the HC group. IOD: Integrated Optical Density. Scale bar: 200x = 50 *μ*m, 400x = 20 *μ*m.

To determine the difference in ACLY expression between normal pregnancy and patients with RSA, qRT-PCR and Western blotting were performed using villus tissues. The results showed that ACLY expression in the villi of patients in the RSA group was significantly lower than that in the HC group at the mRNA and protein levels (Figures 2(a) and 2(b)). In addition, we performed immunohistochemical staining on villi to further verify the expression of ACLY, and the images were of a 200x or 400x visual field under the microscope. The results showed that ACLY levels in villi of patients in the RSA group were significantly lower than those in the HC group (Figure 2(c)). These results suggest that ACLY expression is downregulated in villi of patients with RSA.

### 3.3. Construction of *ACLY* Knockdown Trophoblast Cell Line

**Figure 3:**
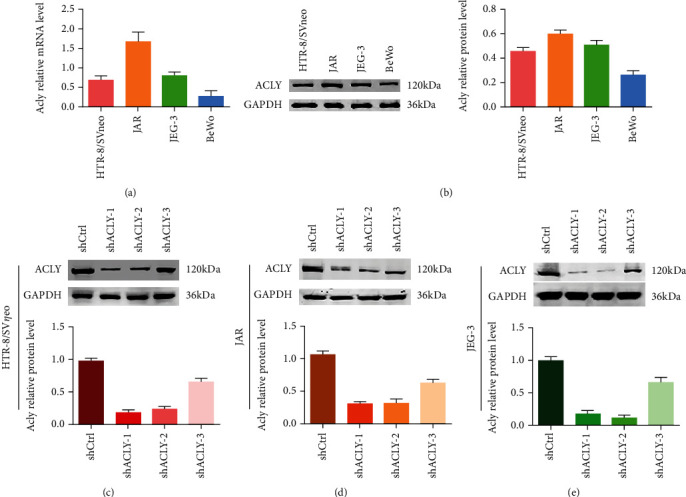
Construction of the *ACLY* knockdown trophoblast line. (a) The expression of *ACLY* in four trophoblastic cell lines, as detected using qRT-PCR. (b) The protein level of ACLY in four trophoblastic cell lines, as detected using Western blotting. (c–e) The protein level of ACLY in HTR-8, JEG-3, and JAR cells, as detected using Western blotting.

The biological dysfunction of trophoblasts is closely related to the pathogenesis of RSA; therefore, we first observed the expression of ACLY in HTR-8, JEG-3, JAR, and BeWo trophoblast lines using qRT-PCR and Western blotting. The results showed that among the four cell lines, the expression of ACLY in BeWo cells was the lowest and was expressed at higher levels in HTR-8, JEG-3, and JAR cells (Figures 3(a) and 3(b)). Subsequently, we used three different sequences to knock down the *ACLY* genes in HTR-8, JEG-3, and JAR cells and then detected the knockdown efficiency using Western blotting. The results showed that the ACLY protein level in the shACLY-1 and shACLY-2 group was significantly reduced (Figures 3(c)–3(e)).

### 3.4. Knockdown of *ACLY* in Trophoblasts Inhibited M2 Polarization of THP-1 Macrophages

**Figure 4:**
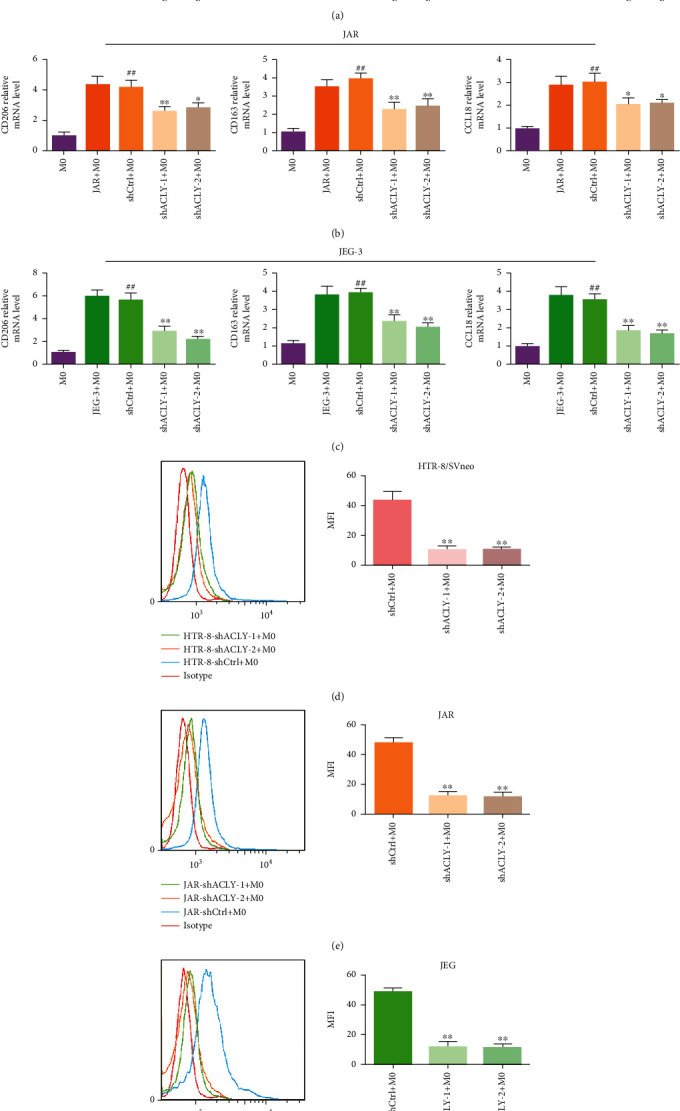
Knockdown of *ACLY* in HTR-8, JAR, and JEG-3 cells inhibited M2 polarization. (a–c) The expression of genes encoding M2 macrophage markers (CD163, CD206, and CCL18) in M0 macrophages and cocultured macrophages, as detected using qRT-PCR. (d–f) Mean fluorescence intensity (MFI) of CD206, as analyzed using flow cytometry in the three cell lines. ^∗^*p* < 0.05, ^∗∗^*p* < 0.01, compared with shCtrl+M0; ^##^*p* < 0.01, compared with M0.

The functional distortion of decidual macrophages is closely related to the occurrence of RSA. In addition, there is crosstalk and interaction between trophoblast cells and decidual macrophages. To explore whether *ACLY* knockdown in trophoblasts affected the polarization of THP-1 macrophages, we selected shACLY-1 and shACLY-2 to knockdown the ACLY genes in HTR-8, JEG-3, and JAR cells. We transfected HTR-8, JAR, and JEG-3 cells with shRNA *ACLY* or empty vector virus. Then, THP-1 macrophages were cultured in the conditioned medium of the transfected cells for 72 hours. qRT-PCR was then used to detect the expression of M2 macrophage markers *CD206*, *CD163*, and *CCL18*. The results showed that compared with the M0 group, the mRNA levels of M2 macrophage markers *CD206*, *CD163*, and *CCL18* were upregulated after treatment with HTR-8/JAR/JEG-3-shctrl supernatant, while their mRNA levels were downregulated significantly after treatment with HTR-8, JAR, or JEG-3-shACLY-1/-2 supernatant (Figures 4(a)–4(c)). Moreover, flow cytometry analysis showed that the mean fluorescence intensity (MFI) of CD206 in the shACLY-1/-2 group was decreased compared with that in the shCtrl group in the three cell lines (Figures 4(d)–4(f)). These results suggested that a lack of ACLY in trophoblasts inhibited macrophage polarization to M2.

### 3.5. Trophoblast-Derived IL-10 Promotes M2 Polarization

**Figure 5:**
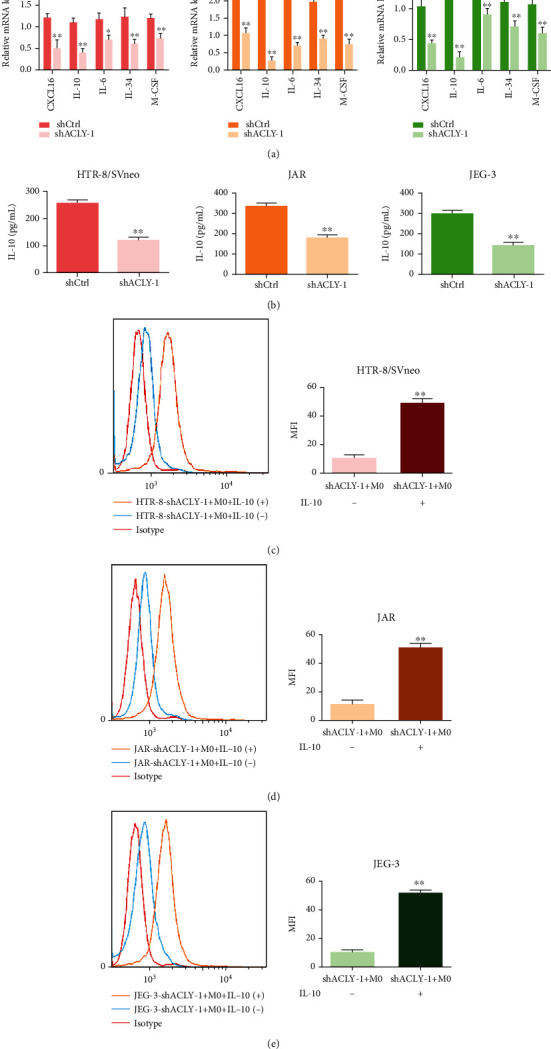
The trophoblast-derived cytokine IL-10 promotes M2 polarization. (a) The expression of genes encoding trophoblast-derived cytokines (IL-6, IL-10, CXCL16, M-CSF, and IL-34) that regulate macrophage M2 polarization, as detected using qRT-PCR. (b) The protein level of IL-10, as detected using ELISA. (c–e) The mean fluorescence intensity (MFI) of CD206, as analyzed using flow cytometry. ^∗^*p* < 0.05, ^∗∗^*p* < 0.01, compared with shCtrl or shACLY+M0 + IL-10(-). After M0 macrophages were treated with HTR-8, JAR, or JEG-3-shACLY supernatant, with or without IL-10 (50 ng/ml), the MFI of CD206 was analyzed using flow cytometry.

Studies have shown that trophoblasts can transmit signals to decidual macrophages through secreted factors. To explore the potential mechanism, we first analyzed the mRNA expression of cytokines related to trophoblast-derived regulation of macrophage M2 polarization (IL-6, IL-10, CXCL16, M-CSF, and IL-34) using qRT-PCR. The results showed that compared with the control group (shCtrl), *IL6*, *IL10*, *CXCL16*, *MCSF*, and *IL34* in HTR-8, JAR, and JEG-3 cells transfected with shRNA ACLY virus decreased by varying degrees (Figure 5(a)). Studies have shown that IL-10 was closely related to epigenetic modifications [18]; therefore, we further detected the level of IL-10 using ELISA. The results showed that IL-10 levels were significantly reduced in HTR-8, JAR, and JEG-3 cells transfected with shRNA *ACLY* virus (Figure 5(b)), which was consistent with the results of qRT-PCR. Therefore, we focused on the role of IL-10. After M0 macrophages were treated with HTR-8, JAR, or JEG-3-shACLY-1 supernatant, with or without IL-10 (50 ng/ml), the average fluorescence intensity of CD206 was analyzed using flow cytometry. The results showed that the MFI of CD206 increased after adding IL-10, which promoted M2 polarization (Figures 5(c)–5(e)). These results suggest that trophoblast-derived cytokine IL-10 is involved in M2 polarization.

### 3.6. *ACLY* Knockdown Leads to Changes in IL-10 Expression through Histone Acetylation

**Figure 6:**
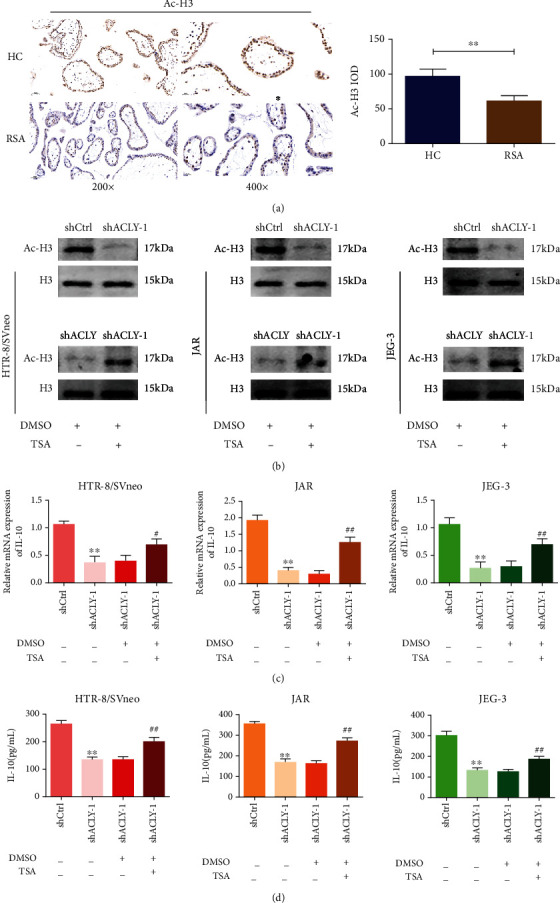
Downregulation of *ACLY* leads to change in IL-10 expression through histone acetylation. (a) The protein levels of acetylated histone H3 in villi of patients with the RSA and the HC groups, as measured using immunohistochemistry (*n* = 5). (b) The levels of total acetylated histone H3 and histone H3, as detected using Western blotting. (c) The expression of *IL10*, as detected using qRT-PCR. (d) The protein level of IL-10, as detected using ELISA. ^∗∗^*p* < 0.01, compared with the HC group; ^∗∗^*p* < 0.01, compared with shCtrl; ^#^*p* < 0.05, ^##^*p* < 0.01, compared with shACLY. IOD: Integrated Optical Density. Scale bar: 200x = 50 *μ*m, 400x = 20 *μ*m.

ACLY is very important for the epigenetic regulation of histone acetylation. First, we performed immunohistochemical staining on villi to verify the expression of H3 acetylation, and the images were of a 200x or 400x visual field under the microscope. It showed that histone H3 acetylation in villi of patients in the RSA group was significantly lower than those in the HC group (Figure 6(a)) These results suggest that H3 acetylation is downregulated in villi of patients with RSA. To further clarify the potential mechanism of ACLY, we detected histone H3 acetylation in trophoblasts. The results showed that compared with that in the shCtrl group, histone H3 acetylation in HTR-8, JAR, and JEG-3 cells transfected with shRNA *ACLY* virus decreased by varying degrees but did not affect the total level of H3. Subsequently, treatment with the histone deacetylase inhibitor trichostatin A (TSA) rescued the histone H3 acetylation level (Figure 6(b)). Considering the effect of ACLY on the expression of trophoblast-derived IL-10, we further studied whether ACLY regulates the expression of cytokine IL-10 by regulating histone acetylation. The results of qRT-PCR and ELISA consistently showed that IL-10 level decreased significantly in HTR-8, JAR, and JEG-3 cells transfected with shRNA *ACLY* virus, and downregulation of IL-10 could be restored after TSA treatment (Figures 6(c) and 6(d)). It suggests that a lack of ACLY in trophoblasts may inhibit IL-10 expression by inhibiting histone acetylation.

### 3.7. Endoplasmic Reticulum Stress Is Involved in IL-10-Mediated Macrophage Polarization

**Figure 7:**
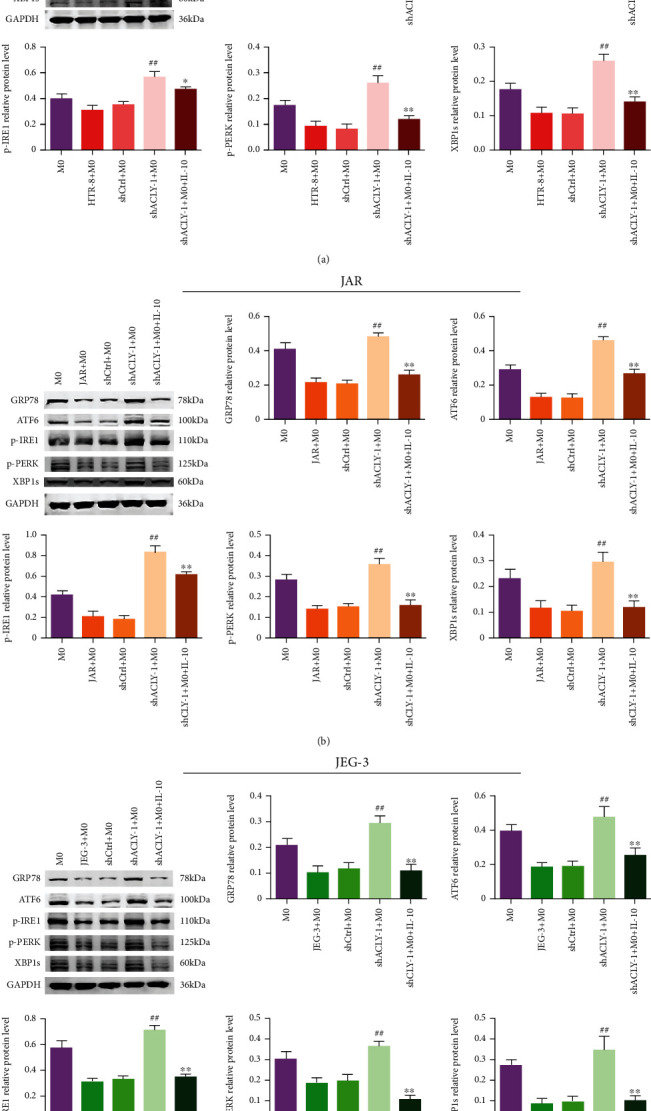
Endoplasmic reticulum stress is involved in IL-10-mediated macrophage polarization. (a–c) The levels of endoplasmic reticulum stress-related proteins, as detected using Western blotting (final concentration of IL-10 was 50 ng/ml). ^##^*p* < 0.01, compared with shCtrl+M0; ^∗^*p* < 0.05, ^∗∗^*p* < 0.01, compared with shACLY+M0.

**Figure 8:**
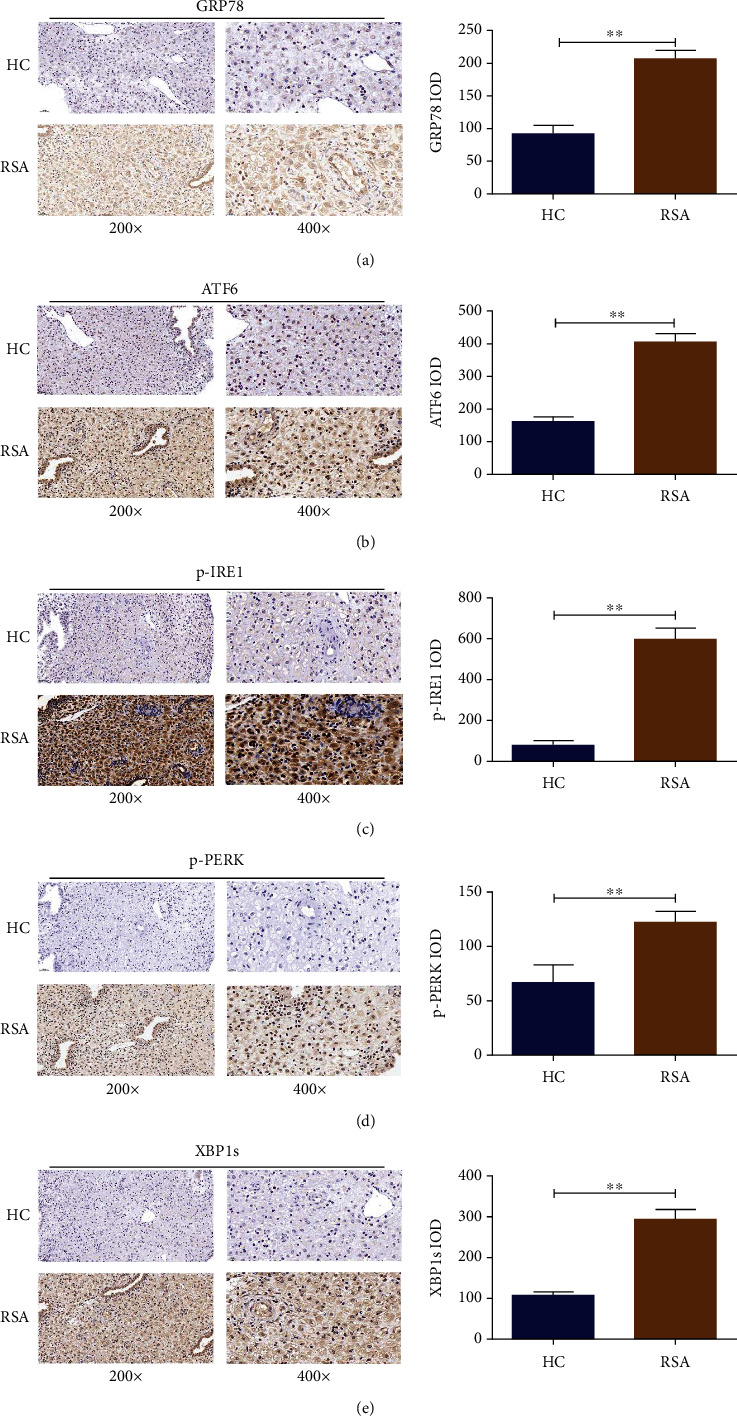
(a–e) The protein levels of endoplasmic reticulum stress-related proteins in decidua of patients with the RSA and the HC groups, as measured using immunohistochemistry (*n* = 5). ^∗∗^*p* < 0.01, compared with the HC group. IOD: Integrated Optical Density. Scale bar: 200x = 50 *μ*m, 400x = 20 *μ*m.

Studies have shown that endoplasmic reticulum stress is involved in the process of M2 polarization of macrophages. To explore the internal mechanism, we treated M0 macrophages with HTR-8, JAR, or JEG-3 supernatant, with or without IL-10 (50 ng/ml) and detected the levels of endoplasmic reticulum stress-related proteins in macrophages using Western blotting. The results showed that compared with the HTR-8, JAR, and JEG-3-shCtrl groups, the level of endoplasmic reticulum stress-related proteins XBP1, p-PERK, p-IRE1, ATF6, and GRP78 in macrophages increased significantly after HTR-8, JAR, or JEG-3-shACLY-1 supernatant treatment; however, the levels of endoplasmic reticulum stress-related proteins in macrophages were decreased after IL-10 addition (Figures 7(a)–7(c)). Then, we performed immunohistochemical staining to detected the levels of endoplasmic reticulum stress-related proteins XBP1, p-PERK, p-IRE1, ATF6, and GRP78 in decidua from normal pregnancy and patients with RSA (Figures 8(a)–8(e)), and the results were consistent with those of Western blotting. The above results suggested that inhibition of endoplasmic reticulum stress mediated by trophoblast-derived IL-10 might be involved in macrophage polarization.

## 4. Discussion

**Figure 9:**
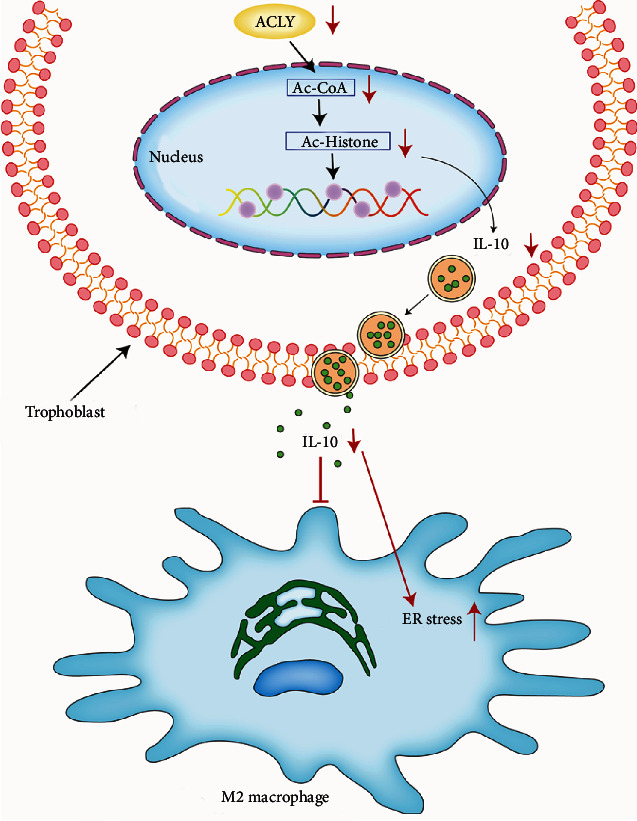
ACLY is decreased in placental villi from RSA, which may lead to the inhibition of histone acetylation in trophoblasts, thereby reducing the secretion of IL-10. Reduced IL-10 secretion activates endoplasmic reticulum stress in macrophages, thus inhibiting their M2 polarization.

The present study demonstrated that ACLY was highly expressed in placental villi and extravillous trophoblasts of normal pregnancy but was downregulated in the placental villi of RSA. Knockdown of *ACLY* in trophoblasts reduced the secretion of IL-10 by inhibiting histone acetylation. The reduced secretion of IL-10 by trophoblasts activated endoplasmic reticulum stress in macrophages, inhibiting their polarization to the M2 phenotype (Figure 9).

RSA, defined as two or more consecutive abortions, affects 5% of women of childbearing age [19]. The causes of RSA are complex, including genetic factors, abnormal female anatomy, hormones, infection, and mental health [20]. The fetus can be regarded as a semiallogeneic graft implanted into the mother. In a successful pregnancy, the mother will not reject the fetus because of the immune tolerance mechanism at the maternal-fetal interface [21]. The generation of maternal-fetal immune tolerance is very important for the successful establishment of mammalian pregnancies. Failure of maternal-fetal immune tolerance might lead to abnormal pregnancy diseases, including RSA [22–24]. The maternal-fetal interface comprises of a series of immune cells, such as decidual natural killer (dNK) cells, macrophages, T cells, dendritic cells, and B cells [25, 26]. In addition to these immune cells, fetal-derived trophoblast cells invade the maternal myometrium, which is also essential for maintaining immune tolerance [27, 28]. Decidual macrophages are in close contact with trophoblast cells during placental development, and the relationship between them is very important to establish and maintain a healthy pregnancy [1].

ATP citrate lyase (ACLY), an enzyme that generates acetyl CoA from citric acid, is the first rate control enzyme responsible for lipid synthesis and connects cell metabolism with histone acetylation [29]. Acetyl CoA is a key metabolic intermediate connecting metabolism, signal transduction, and epigenetics. It is an acetyl donor for protein acetylation reactions and plays an important role in chromatin dynamics and gene regulation [30, 31]. Many studies have established a close relationship between epigenetic regulation and placental trophoblast function by proving the key role of epigenetic regulatory factors in maintaining a healthy pregnancy [32, 33]. Disorder of ACLY is related to various diseases, including cancer [34]. Recent studies have shown that ACLY-mediated metabolic recombination is essential for epigenetic regulation of macrophage activation through histone acetylation [35, 36]; however, the role of ACLY in the maternal-fetal interface is unclear. Our results showed that ACLY was expressed positively in cytotrophoblasts (CTBs), syncytiotrophoblasts (STBs), and extravillous trophoblasts (EVTs). Interestingly, we found that ACLY was also expressed in other cells in decidua, and their existence may have a certain pathophysiological effect, but we think it has nothing to do with the theme of this study. Moreover, we found that ACLY was significantly reduced in the villous tissue in the RSA group. These results suggested that the decrease in the ACLY level in villous trophoblasts is associated with RSA. Therefore, we detected the expression level of ACLY in four trophoblast cell lines (HTR-8, JEG-3, JAR, and BeWo), which showed that ACLY was highly expressed in HTR-8, JAR, and JEG-3 cell lines relative to that in BeWo cells. To clarify the function of ACLY, we knocked down the *ACLY* gene in HTR-8, JEG-3, and JAR cells for further experiments.

Macrophages are the second most abundant immune cell group in the pregnant uterus, accounting for about 20% of the total number of decidual leukocytes [37]. Macrophages can be divided into two polarization types according to their phenotype and secreted cytokines, namely, classically activated M1 macrophages and selectively activated M2 macrophages [38]. Macrophage polarization plays an important role in immune tolerance at the maternal-fetal interface. A change in macrophage polarization can lead to adverse pregnancy outcomes, such as infertility, RSA, and preterm birth [39]. Macrophages in the maternal-fetal interface are mainly M2 type, secreting high level of inhibitory cytokine IL-10 and low level of proinflammatory cytokine IL-1*β*, which is conducive to maintaining immune tolerance and normal pregnancy [40]. In maternal-fetal crosstalk, fetal trophoblast cells can secrete a variety of molecules to regulate immune tolerance, such as cytokines and chemokines [41, 42]. In addition, trophoblast cells can regulate the behavior of decidual macrophages by secreting cytokines or chemokines and participate in the induction of decidual macrophages into the M2 phenotype [43]. To investigate whether ACLY knockdown in trophoblasts is involved in the polarization of THP-1 macrophages, HTR-8, JAR, and JEG-3 cells were transfected with shRNA ACLY or empty vector virus. M0 macrophages were then incubated in conditioned media from HTR-8, JAR, or JEG cells transfected with shCtrl and HTR-8, JAR, or JEG cells transfected with shACLY for 72 hours. Untreated M0 macrophages were used as the control group. Our results showed that impaired ACLY expression in trophoblasts inhibited the M2 polarization of macrophages.

Trophoblasts can transmit signals to decidual macrophages through trophoblast secretory factors [44]. Interesting findings reported by Wang et al. [45] that trophoblast-derived CXCL16 induces M2 macrophage polarization that in turn inactivates NK cells at the maternal-fetal interface. Svensson-Arvelund et al. [46] found that that the human fetal placenta itself, particularly through trophoblast cells, was able to create a homeostatic and tolerant environment by producing soluble factors (M-CSF, IL-10, TGF-*β*, and TRAIL) that induced the polarization of homeostatic macrophages and the expansion of Tregs, as well as limited excessive Th cell activation. Recently, Ding et al. [47] found that trophoblast-derived IL-6 served as an important factor for normal pregnancy by activating Stat3-mediated M2 macrophage polarization. Besides, IL-34, a newly discovered cytokine, was present at the fetal-maternal interface and induced immunoregulatory macrophages of a decidual phenotype in vitro [48]. Therefore, to explore the potential mechanism, we first analyzed the expression of trophoblast-derived cytokines (IL-6, IL-10, CXCL16, M-CSF, and IL-34) in trophoblast cell lines. We found that compared with the control group (shCtrl), IL6, IL10, CXCL16, MCSF, and IL34 in HTR-8, JAR, and JEG-3 cells transfected with shRNA ACLY virus decreased by varying degrees.

ACLY produces acetyl CoA, which provides a donor substrate for histone acetylation [49]. In addition, histone acetylation is important for the expression of cytokines [50, 51]. Studies have shown that IL-10 was closely related to epigenetic modifications [18]. In addition, changes in histone modifications, especially histone acetylation, can lead to abnormal expression of IL-10 mRNA [52, 53]. Therefore, we focused on the role of IL-10. It showed that trophoblast-derived cytokine IL-10 was involved in M2 polarization. And we further explored whether the changes in IL-10 secretion caused by ACLY deletion were related to histone acetylation. The results showed that a lack of ACLY in trophoblasts might reduce the expression of IL-10 by inhibiting histone acetylation. A previous study showed that changes in histone modification, especially histone acetylation, can lead to abnormal expression of *IL10* mRNA [18], which is consistent with our results.

The endoplasmic reticulum (ER) is an important intracellular organelle responsible for protein synthesis, folding, and modification; lipid synthesis; and calcium storage [54]. The unfolded protein response (UPR) is an adaptive intracellular signaling pathway that responds to ER stress by weakening overall protein translation and degrading unfolded proteins [55]. Hypersecretory cell types, such as immune cells, might be highly sensitive to ER stress because of their required increased protein synthesis and folding [56]. For example, a study found IRE1*α* ablation enhanced M2 polarization of macrophages in a cellular autonomous manner [57]. Other studies have shown that the activation of ER stress plays a role in inhibiting the M2 polarization of macrophages [58]. These results suggest that the polarization of M2 macrophages is closely related to their ER stress state. Therefore, after using the coculture system, we detected the expression of ER stress marker proteins such as p-PERK, p-IRE1, ATF6, and GRP78 in macrophages. The results showed that compared with the HTR-8, JAR, and JEG-3-shCtrl groups, the ER stress marker proteins of macrophages increased significantly after culture with the supernatants of HTR-8, JAR, or JEG-3-shACLY. Adding IL-10 could reverse the increase in ER stress marker proteins in macrophages caused by *ACLY* knockdown in trophoblasts. This is consistent with previous research results [58].

In conclusion, our data demonstrated the important role of ACLY in the pathogenesis of RSA. ACLY was strongly expressed in the placental villi and extravillous trophoblasts of normal pregnancy but was downregulated in the placental villi of RSA. The knockdown of *ACLY* in trophoblasts reduced the secretion of IL-10 by inhibiting histone acetylation. The reduced secretion of IL-10 by trophoblasts caused activation of ER stress of macrophages, which inhibited the polarization of macrophages to M2 macrophages. Therefore, ACLY targeting acetyl CoA production might be a potential therapeutic intervention for RSA. The epigenetic effect of ACLY on RSA is very important and cannot be ignored. However, there are still some limitations in this study, as further in vivo investigations is warranted in the future. In-depth studies relating to the regulation of the ACLY on trophoblasts will help us to elucidate the pathogenesis of a class of primary trophoblastic diseases such as RSA and provide new therapeutic targets for the treatment of RSA.

## Figures and Tables

**Table 1:** Demographic and clinical characteristics of the study population.

Characteristics	HC	RSA	*p* value
Age (years)	30.04 ± 3.03	30.36 ± 2.41	0.681
BMI (kg/m^2^)	21.81 ± 1.49	22.09 ± 1.72	0.536
Gestation age (weeks)	7.32 ± 0.80	7.44 ± 1.08	0.658
Number of miscarriages	0.12 ± 0.33	2.76 ± 0.72	< 0.001

HC: healthy control; RSA: recurrent spontaneous abortion; BMI: body mass index.

## Data Availability

The original data is available upon reasonable request.

## References

[1] Mor G., Abrahams V. M. (2003). Potential role of macrophages as immunoregulators of pregnancy. *Reproductive Biology and Endocrinology*.

[2] Bulmer J. N., Williams P. J., Lash G. E. (2010). Immune cells in the placental bed. *The International Journal of Developmental Biology*.

[3] Renaud S. J., Postovit L. M., Macdonald-Goodfellow S. K., McDonald G. T., Caldwell J. D., Graham C. H. (2005). Activated macrophages inhibit human cytotrophoblast invasiveness in vitro. *Biology of Reproduction*.

[4] Mills C. D., Kincaid K., Alt J. M., Heilman M. J., Hill A. M. (2000). M-1/M-2 macrophages and the Th1/Th2 paradigm. *Journal of Immunology*.

[5] Laskin D. L., Sunil V. R., Gardner C. R., Laskin J. D. (2011). Macrophages and tissue injury: agents of defense or destruction?. *Annual Review of Pharmacology and Toxicology*.

[6] Gordon S., Martinez F. O. (2010). Alternative activation of macrophages: mechanism and functions. *Immunity*.

[7] Amara A. B., Gorvel L., Baulan K. (2013). Placental macrophages are impaired in chorioamnionitis, an infectious pathology of the placenta. *Journal of Immunology*.

[8] Tsao F. Y., Wu M. Y., Chang Y. L., Wu C. T., Ho H. N. (2018). M1 macrophages decrease in the deciduae from normal pregnancies but not from spontaneous abortions or unexplained recurrent spontaneous abortions. *Journal of the Formosan Medical Association*.

[9] Xu L., Li Y., Sang Y., Li D. J., Du M. (2021). Crosstalk between trophoblasts and decidual immune cells: the cornerstone of maternal-fetal immunotolerance. *Frontiers in Immunology*.

[10] Sica A., Mantovani A. (2012). Macrophage plasticity and polarization: in vivo veritas. *The Journal of Clinical Investigation*.

[11] Guenther S., Vrekoussis T., Heublein S. (2012). Decidual macrophages are significantly increased in spontaneous miscarriages and over-express FasL: a potential role for macrophages in trophoblast apoptosis. *International Journal of Molecular Sciences*.

[12] Dominguez M., Brune B., Namgaladze D. (2021). Exploring the role of ATP-citrate lyase in the immune system. *Frontiers in Immunology*.

[13] Arias-Sosa L. A., Acosta I. D., Lucena-Quevedo E., Moreno-Ortiz H., Esteban-Perez C., Forero-Castro M. (2018). Genetic and epigenetic variations associated with idiopathic recurrent pregnancy loss. *Journal of Assisted Reproduction and Genetics*.

[14] Rull K., Nagirnaja L., Laan M. (2012). Genetics of recurrent miscarriage: challenges, current knowledge, future directions. *Frontiers in Genetics*.

[15] Pietropaolo V., Prezioso C., Moens U. (2021). Role of virus-induced host cell epigenetic changes in cancer. *International Journal of Molecular Sciences*.

[16] Munro S. K., Balakrishnan B., Lissaman A. C., Gujral P., Ponnampalam A. P. (2021). Cytokines and pregnancy: potential regulation by histone deacetylases. *Molecular Reproduction and Development*.

[17] Mishra N., Reilly C. M., Brown D. R., Ruiz P., Gilkeson G. S. (2003). Histone deacetylase inhibitors modulate renal disease in the MRL-lpr/lpr mouse. *The Journal of Clinical Investigation*.

[18] Zheng Z., Huang G., Gao T. (2020). Epigenetic changes associated with interleukin-10. *Frontiers in Immunology*.

[19] Williams Z. (2012). Inducing tolerance to pregnancy. *The New England Journal of Medicine*.

[20] Li T. C., Makris M., Tomsu M., Tuckerman E., Laird S. (2002). Recurrent miscarriage: aetiology, management and prognosis. *Human Reproduction Update*.

[21] Li W. X., Xu X. H., Jin L. P. (2021). Regulation of the innate immune cells during pregnancy: an immune checkpoint perspective. *Journal of Cellular and Molecular Medicine*.

[22] Trowsdale J., Betz A. G. (2006). Mother’s little helpers: mechanisms of maternal-fetal tolerance. *Nature Immunology*.

[23] Leber A., Zenclussen M. L., Teles A. (2011). Pregnancy: tolerance and suppression of immune responses. *Methods in Molecular Biology*.

[24] Tao Y., Li Y. H., Zhang D. (2021). Decidual CXCR4+CD56brightNK cells as a novel NK subset in maternal–foetal immune tolerance to alleviate early pregnancy failure. *Clinical and Translational Medicine*.

[25] Yang F., Zheng Q., Jin L. (2019). Dynamic function and composition changes of immune cells during normal and pathological pregnancy at the maternal-fetal interface. *Frontiers in Immunology*.

[26] Faas M. M., de Vos P. (2017). Uterine NK cells and macrophages in pregnancy. *Placenta*.

[27] Arck P. C., Hecher K. (2013). Fetomaternal immune cross-talk and its consequences for maternal and offspring’s health. *Nature Medicine*.

[28] Wu X., Jin L. P., Yuan M. M., Zhu Y., Wang M. Y., Li D. J. (2005). Human first-trimester trophoblast cells recruit CD56brightCD16- NK cells into decidua by way of expressing and secreting of CXCL12/stromal cell-derived factor 1. *Journal of Immunology*.

[29] Wen J., Min X., Shen M. (2019). ACLY facilitates colon cancer cell metastasis by CTNNB1. *Journal of Experimental & Clinical Cancer Research*.

[30] Pietrocola F., Galluzzi L., Bravo-San Pedro J. M., Madeo F., Kroemer G. (2015). Acetyl coenzyme A: a central metabolite and second messenger. *Cell Metabolism*.

[31] Zheng W., Tasselli L., Li T. M., Chua K. F. (2021). Mammalian SIRT6 represses invasive cancer cell phenotypes through ATP citrate lyase (ACLY)-dependent histone acetylation. *Genes (Basel)*.

[32] Kohan-Ghadr H. R., Kadam L., Jain C., Armant D. R., Drewlo S. (2016). Potential role of epigenetic mechanisms in regulation of trophoblast differentiation, migration, and invasion in the human placenta. *Cell Adhesion & Migration*.

[33] Hemberger M. (2007). Epigenetic landscape required for placental development. *Cellular and Molecular Life Sciences*.

[34] Icard P., Wu Z., Fournel L., Coquerel A., Lincet H., Alifano M. (2020). ATP citrate lyase: a central metabolic enzyme in cancer. *Cancer Letters*.

[35] Lauterbach M. A., Hanke J. E., Serefidou M. (2019). Toll-like receptor signaling rewires macrophage metabolism and promotes histone acetylation via ATP-citrate lyase. *Immunity*.

[36] Covarrubias A. J., Aksoylar H. I., Yu J. (2016). Akt-mTORC1 signaling regulates Acly to integrate metabolic input to control of macrophage activation. *eLife*.

[37] Erlebacher A. (2013). Immunology of the maternal-fetal interface. *Annual Review of Immunology*.

[38] Biswas S. K., Chittezhath M., Shalova I. N., Lim J. Y. (2012). Macrophage polarization and plasticity in health and disease. *Immunologic Research*.

[39] Brown M. B., von Chamier M., Allam A. B., Reyes L. (2014). M1/M2 macrophage polarity in normal and complicated pregnancy. *Frontiers in Immunology*.

[40] Nagamatsu T., Schust D. J. (2010). The immunomodulatory roles of macrophages at the maternal-fetal interface. *Reproductive Sciences*.

[41] Ren L., Liu Y. Q., Zhou W. H., Zhang Y. Z. (2012). Trophoblast-derived chemokine CXCL12 promotes CXCR4 expression and invasion of human first-trimester decidual stromal cells. *Human Reproduction*.

[42] Sun Y., Wu S., Zhou Q., Li X. (2021). Trophoblast-derived interleukin 9 mediates immune cell conversion and contributes to maternal-fetal tolerance. *Journal of Reproductive Immunology*.

[43] Sun F., Wang S., Du M. (2021). Functional regulation of decidual macrophages during pregnancy. *Journal of Reproductive Immunology*.

[44] Aldo P. B., Racicot K., Craviero V., Guller S., Romero R., Mor G. (2014). Trophoblast induces monocyte differentiation into CD14+/CD16+ macrophages. *American Journal of Reproductive Immunology*.

[45] Wang X. Q., Zhou W. J., Hou X. X., Fu Q., Li D. J. (2018). Trophoblast-derived CXCL16 induces M2 macrophage polarization that in turn inactivates NK cells at the maternal-fetal interface. *Cellular & Molecular Immunology*.

[46] Svensson-Arvelund J., Mehta R. B., Lindau R. (2015). The human fetal placenta promotes tolerance against the semiallogeneic fetus by inducing regulatory T cells and homeostatic M2 macrophages. *Journal of Immunology*.

[47] Ding J., Yang C., Cheng Y. (2021). Trophoblast-derived IL-6 serves as an important factor for normal pregnancy by activating Stat3-mediated M2 macrophages polarization. *International Immunopharmacology*.

[48] Lindau R., Mehta R. B., Lash G. E. (2018). Interleukin-34 is present at the fetal-maternal interface and induces immunoregulatory macrophages of a decidual phenotype in vitro. *Human Reproduction*.

[49] Guo Q., Kang H., Wang J. (2021). Inhibition of ACLY leads to suppression of osteoclast differentiation and function via regulation of histone acetylation. *Journal of Bone and Mineral Research*.

[50] Rauen T., Hedrich C. M., Tenbrock K., Tsokos G. C. (2013). cAMP responsive element modulator: a critical regulator of cytokine production. *Trends in Molecular Medicine*.

[51] Kiguchi N., Kobayashi Y., Saika F., Kishioka S. (2013). Epigenetic upregulation of CCL2 and CCL3 via histone modifications in infiltrating macrophages after peripheral nerve injury. *Cytokine*.

[52] Duan L., Yi M., Chen J., Li S., Chen W. (2016). Mycobacterium tuberculosis EIS gene inhibits macrophage autophagy through up-regulation of IL-10 by increasing the acetylation of histone H3. *Biochemical and Biophysical Research Communications*.

[53] Mukherjee S., Mukherjee B., Mukhopadhyay R. (2014). Imipramine exploits histone deacetylase 11 to increase the IL-12/IL-10 ratio in macrophages infected with antimony-resistant Leishmania donovani and clears organ parasites in experimental infection. *Journal of Immunology*.

[54] Schwarz D. S., Blower M. D. (2016). The endoplasmic reticulum: structure, function and response to cellular signaling. *Cellular and Molecular Life Sciences*.

[55] Ron D., Walter P. (2007). Signal integration in the endoplasmic reticulum unfolded protein response. *Nature Reviews. Molecular Cell Biology*.

[56] Soto-Pantoja D. R., Wilson A. S., Clear K. Y., Westwood B., Triozzi P. L., Cook K. L. (2017). Unfolded protein response signaling impacts macrophage polarity to modulate breast cancer cell clearance and melanoma immune checkpoint therapy responsiveness. *Oncotarget*.

[57] Shan B., Wang X., Wu Y. (2017). The metabolic ER stress sensor IRE1*α* suppresses alternative activation of macrophages and impairs energy expenditure in obesity. *Nature Immunology*.

[58] Minton K. (2017). Immunometabolism: Stress-induced macrophage polarization. *Nature Reviews Immunology*.

